# Step up for wellness: Reducing sedentary lifestyles for a healthier future: Comment on "Intervening to reduce sedentary behavior among African American elders: the "Stand Up and Move More" intervention"

**DOI:** 10.34172/hpp.025.43861

**Published:** 2025-05-06

**Authors:** Priyanga Chandrasekaran, Priyadharshini Ragavane, Roshni Maria Irwina A

**Affiliations:** Department of Public Health Dentistry, Indira Gandhi Institute of Dental Sciences, Sri Balaji Vidyapeeth University, Puducherry, India

 We recently reviewed your article, *“Intervening to reduce sedentary behavior among African American elders: the ‘stand up and move more’ intervention,”*^1^ and found it to be a compelling strategy to address sedentary behavior in a high-risk population. The intervention’s community-based, culturally tailored approach is particularly noteworthy. By promoting small, manageable behavior changes like standing more and incorporating light physical activities, it has resulted in impressive improvements in physical function, balance, and quality of life for participants.

 One limitation worth addressing is the intervention’s focus on female participants, which restricts its broader applicability. Future research should aim for greater diversity, ensuring that the findings can be generalized across genders and other demographic groups. The SUMM model, however, aligns perfectly with global public health priorities, particularly in promoting healthy aging by reducing sedentary behavior. Its adaptability makes it a powerful tool for diverse populations, especially those undergoing urbanization and cultural shifts.^1^

 Although the SUMM intervention has shown promise, the modest reductions in sedentary time suggest there is room for further innovation. Integrating digital tools for self-monitoring could provide the necessary support to sustain long-term behavior changes. We would like to recommend DIGITAL DETOX programs, screen time management, and offline activities essential for combating the harmful effects of excessive screen use, which is often intertwined with sedentary behavior.^2^

 Globally, initiatives like the Netherlands’ and Denmark’s cycling-friendly policies, Singapore’s active living programs, Canada’s ParticipACTION,^3^ India’s Fit India Movement^4^ and the UK’s Daily Mile campaigns^5^ highlight the importance and effectiveness of promoting physical activity for public health. Thereby, the SUMM model can also address both public and planetary health, by encouraging active transportation—walking and cycling—over motorized travel, it helps combat sedentary lifestyles while contributing to environmental sustainability. Integrating active transportation into physical activity guidelines is essential for addressing the twin challenges of health and environmental crises. This approach not only improves public health outcomes but also supports sustainable development by reducing pollution and conserving resources. By aligning such efforts with National Health Policies, we can reduce healthcare burdens, improve quality of life, and empower the elderly. Moving forward, future guidelines should prioritize active transportation, with collaboration across healthcare, transportation, and urban planning sectors, to drive lasting, sustainable change.

## Competing Interests

 None declared.

## Ethical Approval

 Not applicable.
